# Comparing the Accuracy of Osteological Sex Estimation Against Genetic Sex Obtained From Human Dental Calculus in 12th–19th C. British Individuals

**DOI:** 10.1002/ajpa.70328

**Published:** 2026-08-07

**Authors:** Sarah Alice Inskip, Biancamaria Bonucci, Christiana Lyn Scheib, Anna Myfanwy Davies‐Barrett

**Affiliations:** ^1^ School of Heritage and Culture, University of Leicester Leicester UK; ^2^ Estonian Biocentre, Institute of Genomics, University of Tartu Tartu Estonia; ^3^ Department of Zoology University of Cambridge Cambridge UK

**Keywords:** biomolecular analysis, morphological traits, Phenice traits, post‐medieval, sexual dimorphism

## Abstract

**Objectives:**

For anthropologists and forensic scientists, sex estimation using skeletally dimorphic traits is fundamental in biological profile reconstruction. Archaeogenetic research on past populations allows us to identify genetic sex generating opportunities to validate the accuracy of commonly used skeletal traits. We compare genetic sex estimates, derived from aDNA analysis of dental calculus, with macroscopic skeletal observations to ascertain the accuracy of trait‐based estimates on British skeletons.

**Materials and Methods:**

Dental calculus was sampled from 146 individuals from two British sites (Barton‐upon‐Humber and St James's Gardens [London]), and genetic sex was obtained. A combination of frequently analyzed skull and os coxae traits was assessed to determine overall osteological sex; individual skeletal traits were also evaluated separately. These were then compared to genetic sex. A regression analysis of trait scores was used to identify the best trait combination for sex estimation.

**Results:**

We found high agreement between overall osteological sex and genetic sex (97.9%); the os coxa estimate demonstrated almost perfect agreement (97.8%). Skull‐based estimates were less accurate (79.0%). Individual Phenice traits demonstrated the greatest accuracy (80.2%–97.5%), whilst multiple skull traits performed poorly (58.9%–68.8%).

**Discussion:**

Overall, application of osteological sex estimation techniques to British remains was highly accurate, especially using combined traits of the os coxa. However, we identified multiple skeletal features that are not reliable in isolation, but provide new sectioning points and regression equations to improve skull estimate accuracy. We emphasize the need for further work on factors affecting pelvic morphology, and the benefits of dental calculus as aDNA source.

## RESUMEN

**Obiettivi:**

Per antropologi, archeologi e scienziati forensi, la stima del sesso mediante tratti scheletrici dimorfici costituisce una componente fondamentale della ricostruzione del profilo biologico. La ricerca archeogenetica sulle popolazioni del passato consente inoltre di identificare il sesso genetico, offrendo l‘opportunità di verificare l‘accuratezza dei tratti scheletrici comunemente utilizzati. La nostra ricerca confronta le stime del sesso genetico, derivate dall‘analisi del DNA antico (aDNA) del tartaro dentale, con le osservazioni scheletriche macroscopiche, per accertare l‘applicabilità di questi metodi sugli scheletri britannici.

**Materiali e metodi:**

Il tartaro dentale è stato prelevato da 146 individui provenienti da due siti britannici (Barton‐upon‐Humber e St James‘s Gardens (Londra)), e ne è stato determinato il sesso genetico. Per la determinazione morfologica complessiva del sesso, è stata valutata una combinazione di tratti del cranio e dell’osso coxale, spesso usati in ambito archeologico per questo tipo di analisi; inoltre, sono stati valutati separatamente anche i singoli tratti scheletrici. I risultati ottenuti sono stati poi confrontati con le stime di sesso genetico.

**Risultati:**

Abbiamo riscontrato un’elevata concordanza tra il sesso morfologico complessivo complessivo e il sesso genetico (97,8%); l’uso del solo osso coxale ha dimostrato una concordanza quasi perfetta (97,0%). Le stime basate sul cranio sono risultate meno accurate (78,6%), in linea con studi fatti in precedenza. I singoli tratti fenotipici del pube hanno dimostrato la massima accuratezza (97,5‐80,2%), mentre diversi tratti cranici hanno dato risultati scarsi (59,2‐68,3%).

**Discussione:**

Nel complesso, l’applicazione delle tecniche di stima del sesso morfologico ai resti britannici si è rivelata altamente accurata, in particolare utilizzando una combinazione di caratteristiche dell’osso coxale. Tuttavia, abbiamo individuato una serie di caratteristiche scheletriche che, se considerate isolatamente, non sono affidabili, mettendo in evidenza i rischi legati all’utilizzo di un numero troppo esiguo di caratteristiche. Sottolineiamo inoltre la necessità di ulteriori studi volti a valutare i fattori che influenzano la morfologia pelvica, nonché i vantaggi derivanti dall’utilizzo del tartaro dentale come fonte di aDNA.

## 1 Introduction

The accurate estimation of osteological sex in biological anthropology and allied research fields is fundamental for reconstructing demographic and mortality profiles, and quantifying variation between different populations or social groups (Krishan et al. 2016). It is a vital first step in forensic anthropology when dealing with unidentified skeletal remains. Sex estimates are conventionally compiled from the standardized assessment of multiple skeletal morphological traits that vary in expression between biologically female and biologically male individuals. Traits are scored on a scale from “most female” to “most male” and the overall impression provided by the assessment of all available traits results in a “skeletal” or “osteological sex estimation”. This approach is not, however, an exact science, and no traits are binary. While some traits have high accuracy (e.g., Phenice traits of the pubis (Inskip et al. 2019; Lovell 1989; Patriquin et al. 2003; Ubelaker and Volk 2002)), this can differ between populations and time periods (see Table S1). Estimates derived from the combined traits of the skull are demonstrably variable in accuracy, with rates as low as 53.5% (Gupta et al. 2022), and as high as 96.2% (Lewis and Garvin 2016).

The development of forensic medicine has been key in the production of standardized approaches for sex estimation, with the first approach being developed by Krogman (1939). This has inspired the production of multiple standards for sex estimation which are regularly applied across the globe, with perhaps the most well‐known being the WEA (1980), superseded by that outlined by Buikstra and Ubelaker (1994). However, there has been limited systematic testing of the accuracy of these traits on collections other than those that were used to make the methods, especially on non‐contemporary populations (e.g., archaeological). Most accuracy tests have been performed on the Terry or Hamann–Todd collections, from which most of the methods were originally derived, or on other similar modern collections (see Table S1). This can lead to overfitting of the data producing inflated accuracy rates. This is problematic since these methods are used worldwide (Ubelaker and DeGaglia 2017) and perhaps without consideration of global variation in trait expression. Research in forensic settings has highlighted a great deal of variation between contemporary groups of different ethnicities, demonstrating the need for population specific standards (Gupta et al. 2022; Krüger et al. 2015; Patriquin et al. 2003; Tallman 2019). Factors influencing expression could derive from secular change, population change, and variation in health and lifestyle, all of which would equally be at play in the past. Without knowing the accuracy of these traits in populations from different time periods, we cannot be sure that the estimation of sex in archaeological individuals is accurate. More targeted testing on archaeological groups is, therefore, needed to ensure the appropriate use of these traits for sex estimation in different populations.

Ancient DNA (aDNA) studies using archaeological individuals have become more common and accessible in the past 15 years. Identification of chromosomal pairing for the sex chromosomes using genetic analysis provides evidence for the genetic and, subsequently inferred, biological sex of an individual (Skoglund et al. 2013). The comparison of genetic sex with estimated osteological sex can, therefore, provide greater insight into the accuracy of the morphological traits of the skeleton used to infer biological sex in archaeological populations. This can be used to validate and improve the accuracy of macroscopic sex estimations. This approach was utilized by Inskip et al. (2019) on 66 adult individuals from thirteenth to sixteenth century Cambridge, United Kingdom. The agreement between genetic and osteological sex using traits of the pelvis was 91.8%, but only 76.7% when using traits of the skull. In a study of a medieval German sample from Lubeck (*n* = 43), Gupta et al. (2022) found an accuracy of just 53.5% between genetic sex and skull‐based estimates.

Building on Inskip et al. (2019), this paper aims to analyze the accuracy of morphological sex estimates in comparison to genetic sex in a larger sample from two mostly later archaeological British population groups. We aimed to see if similar trends in accuracy rates of individual traits, and sex estimates derived from a combination of traits, were identifiable, and to, hence, evaluate the applicability of commonly used osteological sex estimation traits on late medieval and post‐medieval British groups. Our objectives were to score individual traits commonly used for sex estimation, and to create individual osteological sex estimates by combining the data from traits of the skull, the os coxa, and the skull and os coxa data combined. The osteological sex estimate from each combination of traits was compared to genetic sex as identified from the aDNA recovered from samples of dental calculus, allowing the calculation of accuracy rates for each trait combination. Accuracies for each trait were calculated to identify the best and worst performing traits, to identify potential improvements to future osteological sex estimation approaches. We also assessed the distribution of scores to identify new appropriate sectioning points for “male” and “female,” and produce regression models to identify the best combination of traits to estimate sex. For the first time, we assessed whether side biases existed in the accuracy of individual traits. Furthermore, we show the potential of using dental calculus as a source of human aDNA for sex estimation, which is valuable where destructive sampling of the skeleton is undesirable.

First, we must highlight here what we mean by the different types of sex estimation described in the current research, according to the Sex and Gender Equity in Research (SAGER) guidelines (Heidari et al. 2016). We refer here to biological sex as the female and male categories into which physical and physiological features (including anatomical features and chromosomal genotype) of an individual generally fall. As discussed above, we have ascribed osteological sex based on the morphological assessment of traits of the human skeleton on a scale from most female to most male. Genetic sex was determined from ancient DNA extracted from dental calculus. Sex assignment was based on the relative representation of reads mapping to the X and Y chromosomes, following established methods (Anastasiadou et al. 2024). Individuals with a chromosomal genotype consistent with XX were classified as genetically female, and those consistent with XY were classified as genetically male (see section 3.5). Sex assignments are probabilistic and reflect chromosomal genotype estimates rather than phenotypic sex. While, by necessity, we refer here to categories of male and female, we recognize that biological, osteological and genetic sex are not simply binary constructs (Winter and Birkmann‐Little 2025). We do not refer here to gender—the attribution of the socially‐constructed roles of male, female, or gender‐diverse—which will not necessarily correspond to biological, genetic or osteological sex determinations.

## 2 Individuals Assessed

The current study resulted from research on the Tobacco, Health and History Project, which aimed to assess the impact of tobacco use on health after the intoxicant's arrival into Europe in the sixteenth century. As part of this research, two groups of human skeletal remains from England were assessed for osteological and genetic sex. The sample group consisted of individuals dating to just before and after the introduction of tobacco from the town of Barton‐upon‐Humber, and individuals buried in eighteenth‐nineteenth century central London, from the parish of St James's, Piccadilly (see Table S2 for the breakdown of the samples used for this research). The majority of the remains were post‐medieval (see below). Preservation at both sites was good to excellent; many skeletons exhibited high levels of completeness, making the sample ideal for an investigation of the accuracy of osteological sex traits.

Individuals from Barton were recovered from the church cemetery of St Peter's, which had over 2700 burials, dating from c. 950 to 1855 CE (Waldron 2007). In the current study, 21 individuals from the medieval period (1150–1500 CE) and 44 individuals from the post‐medieval period (1500–1855 CE) were assessed for both osteological and genetic sex. Until the late eighteenth century, most people living in Barton‐upon‐Humber were likely involved in agricultural work (Barton upon Humber Branch Workers' Educational Association 1978, 1980). Diversification of roles in the nineteenth century saw a range of employments within the town, including as laborers, tile, rope and brick makers, shopkeepers, lawyers, and domestic and craft workers, reflecting a range of socioeconomic circumstances (Barton upon Humber Branch Workers' Educational Association 1978).

St James's Gardens Burial Ground was excavated as part of major construction developments between 2017 and 2021. This cemetery, which was an overflow burial ground for the parish of St James's, Piccadilly, was established in 1789 CE, in the area that is now Euston Station, London, and was in use until 1853 CE (Hartle 2022). During this time, thousands of individuals were interred there. The cemetery was divided into four different burial grounds, roughly reflecting socioeconomic standing, from the very poor (paupers' graves) through to the elite of London (Hartle 2022). Individuals were sampled from across the four burial grounds and, as such, the sample reflects a mixed socioeconomic group. The current study assessed 81 individuals from this cemetery for both osteological and genetic sex (see Table S2).

## 3 Methods

### 3.1 Ancient DNA Sampling, Decontamination, Extraction, and Purification

Dental calculus (mineralized dental plaque) is commonly recovered adhering to the teeth of skeletal remains. The lack of modern dental cleaning techniques means this material is often found in abundance on the dentition. Due to its accumulative nature, dental calculus can trap the remains of material from the oral cavity, including particles of consumables, environmental matter, oral bacteria, and, important to this study, endogenous human DNA. This has made it a valuable source of aDNA in forensics studies, as well as in archaeological contexts (Lisman et al. 2023; De La Fuente et al. 2013; Fu et al. 2025). Importantly, in cases of good sample preservation, as in this study, genetic sex estimates using aDNA from calculus can be obtained as an additional step in analyses that mainly focus on reconstructing the oral microbiome, despite the former constituting a small proportion of the total metagenomic signal (Ziesemer et al. 2019; Warinner et al. 2014). This makes it possible to avoid, in certain cases, the need to undertake skeletally destructive sampling from the individual, which highlights the sustainability of ancient dental calculus for archaeological research (Favreau and Patalano 2017).

As there has been shown to be some difference in the composition of the oral microbiome between location on the tooth and tooth position (Gancz et al. 2023), we aimed to sample dental calculus as consistently as possible, although this was highly impacted by the availability of calculus (Table S2). Dental calculus was photographed and recorded for location on the tooth (supra‐/sub‐gingival, mesial, distal, labial/buccal or lingual) and for tooth position and type. The quantity was recorded based on the method provided by Brothwell (1981). Dental calculus was then removed using a metal microspatula, which was cleaned between sampling, at the University of Leicester's Human Remains Laboratory in the School of Heritage and Culture. Samples were placed in a small unused plastic bag/glass vial.

All subsequent steps were performed in the Ancient DNA Facility of the Core Facility of Genomics, Institute of Genomics, University of Tartu (see Text S1).

### 3.2 Molecular Sex Estimation

Genetic sex was estimated for all samples in the study using the script karyo_RxRy_script.py from Anastasiadou et al. (2024). The average coverage of the whole genome for the samples was between 0.0001 and 0.025×. Despite the fact that we were able to estimate the genetic sex on low coverage data, as expected from the original publication, we would like to emphasize that results obtained at coverage levels below 0.005× should be interpreted with caution. The used approach is based on quantifying the number of sequences aligning to an autosomal baseline (Na), defined as the sum of sequences aligning to Chromosome 1 to 22, excluding 13, 18, 21. Afterwards, the metrics Rx and Ry were calculated as, respectively, the proportion between the number of sequences aligning to Chromosome X and Na, and the number of sequences aligning to chromosome Y and Na (see Anastasiadou et al. 2024 for further details).

### 3.3 Osteological Sex Estimation

Six skull features and seven os coxa features were individually examined to estimate osteological sex (Table 1). This includes the nuchal crest (NC), mastoids (MA), supraorbital margin (SM), the glabella (GL), mandibular (mental) eminence (ME), and mandibular shape (MS). These traits were chosen due to frequent usage in osteological sex estimation and their pre‐established reliability as accurate estimators of biological sex. The majority of skull features were recorded based on methods presented by Buikstra and Ubelaker (1994), which scores these traits on a 5‐point scale from 1 (*probable female*), 2 (*possible female*), 3 (*intermediate*), 4 (*possible male*), to 5 (*probable male*). In addition, we used mandibular shape (MS), as presented by Brickley (2004), which scores this feature on a three‐point scale. Following results by Inskip et al. (2019), we did not record frontal bossing or gonial flaring as these traits have previously demonstrated particularly low accuracy rates.

**TABLE 1:** Features of the skull and os coxae macroscopically assessed for osteological sex estimation.

Skeletal region	Trait	Abbreviation	Source
Skull	Nuchal crest	NC	(Buikstra and Ubelaker 1994)
	Mastoid	MA	(Buikstra and Ubelaker 1994)
	Supraorbital margin	SM	(Buikstra and Ubelaker 1994)
	Glabella	GL	(Buikstra and Ubelaker 1994)
	Mandibular eminence	ME	(Buikstra and Ubelaker 1994)
	Mandibular shape	MS	(Brickley 2004)
Os coxae	Ventral arc	VA	(Klales et al. 2012, after Phenice 1969)
	Subpubic concavity	SC	(Klales et al. 2012, after Phenice 1969)
	Ischiopubic ramus ridge	RR	(Klales et al. 2012, after Phenice 1969)
	Subpubic angle	SA	(Brickley 2004; fig. 9a)
	Greater sciatic notch	SN	(Buikstra and Ubelaker 1994)
	Composite arc	CA	(Bruzek 2002)
	Ischiopubic proportions	IP	(Bruzek 2002)

For the os coxa, the traits presented by Phenice (1969)—the ventral arch (VA), ischiopubic ramus ridge (RR), and subpubic concavity (SC) (commonly referred to as the Phenice traits)—were recorded using the adapted method by Klales et al. (2012), which scores these traits on a five‐point scale. The subpubic angle (SA) (Brickley 2004) and greater sciatic notch (SN) (Buikstra and Ubelaker 1994), scored on a five‐point scale, and the composite arch (arc composé, CA) and ischiopubic proportions (IP) (Bruzek 2002), scored on a three‐point scale, were also assessed. We did not include the preauricular sulcus, as this trait has proven highly inaccurate in multiple previous studies (Dee 1981; Gülekon and Turgut 2001; Spring et al. 1989). Traits were assessed individually and independently from other traits to limit confirmation bias.

Using the same five‐point scale, the individual skeletal area (skull or os coxa) was given an estimate based on cumulative evidence from all analyzed traits. Finally, an overall estimate was given based on all observable traits, with observations from the os coxa given priority in cases where there was conflict between estimations of osteological sex based on the skull and the os coxa, as pelvic traits are known to be more reliable in all groups tested so far (see Table S1).

Any trait that was considered unobservable was recorded as such. If more than four of the six skull traits or more than five of the seven pelvic traits were unobservable then that skeletal region was considered unobservable overall. For bilateral traits, at least one side was required to be observable for that trait to be considered present when allocating overall sex estimation for that skeletal region. When combining bilateral traits for logistic regression analysis, the left side was considered the default score, unless unobservable, in which case the score for the right side was used. All traits were recorded by one observer (ADB) and methods were repeated on a subsample of 39 randomly selected individuals to test for intraobserver error. The observer had no osteobiographical knowledge of, or interaction with, the skeletons prior to analysis.

### 3.4 Comparison of aDNA and Osteological Outcomes

General percentage agreement was calculated by the number of osteological sex estimations accurately categorized to be the same as the genetic sex estimation. Individuals considered to be unobservable or intermediate for osteological sex estimation were removed from this calculation. We also present raw accuracies, which include intermediate scores in calculations as incorrectly estimated, following Inskip et al. (2019, 5). This is a valuable statistic, since it highlights the usefulness of the method or trait by showing the proportion of observations that cannot be provided a determining score. For example, a trait that scores as “male” or “female” may highly correlate with genetic sex, but this trait might present as intermediate for a large proportion of the population.

To test agreement between osteological and genetic sex estimates, a Cohen's kappa test was applied to estimates of raw accuracy. This test produces a kappa coefficient (*κ*), which quantifies the level of agreement between two measures. Level of agreement was categorized as: Poor if *κ* < 0.00, Slight if 0.00 ≤ *κ* ≤ 0.20, Fair if 0.21 ≤ *κ* ≤ 0.40, Moderate if 0.41 ≤ *κ* ≤ 0.60, Substantial if 0.61 ≤ *κ* ≤ 0.80, and Almost perfect if *κ* > 0.80, after Watson & Petrie (2010, 1170). Chi‐squared tests explored differences in the accuracy rates between left and right sides, with a significant value suggesting a measurable effect. A *p* value of less than 0.05 was considered to be a significant value. However, we paid attention to values that were less than 0.1.

Following Walker (2008) and Klales et al. (2012), binary logistic regression was employed to produce class membership models for predicting the probability that a certain trait score, or combination of trait scores, will accurately estimate biological sex. Due to the large number of possible trait combinations, backwards step‐wise elimination using likelihood ratios (LR method) was applied to models for the skull and os coxa separately to determine the combination of traits with greatest classification accuracy. This involves running the binary logistic model multiple times, consecutively eliminating the trait that contributes the least to model fit at each step until removal of any more traits will significantly reduce the model fit. This was undertaken using SPSS Statistics 31. Finally, these classification models were tested using the process of leave‐one‐out‐cross‐validation (LOOCV). This process runs the model on the entire sample, bar one (*n* − 1) and is repeated until each individual in the sample has been excluded once. The LOOCV classification accuracy of each model is then calculated as the proportion of correctly classified individuals across all runs. This process was undertaken using R 4.5.3.

As part of the application of binary logistic regression, regression equations for different traits were produced and can be used to provide a probability that an individual is either male or female. These equations provide weights (regression coefficients) to different traits. This allows for a score (or combination of scores) to be input into a calculation, producing a predictive value (*y*), using a sectioning point of 0, where a *y* score of < 0 = female and > 0 = male. The probability that this *y* score (or combination of scores) will indicate a biological female (*pf*) can be calculated using the following equation (where *e* is the base of the natural logarithm, 2.71828):

$$\mathrm{pf}=1/\left(1+e^{-y}\right)$$

The probability that the score (or combination of scores) will indicate a biological male (*pm*) can then be calculated as such:

pm=1−pf

## 4 Results

### 4.1 Human DNA Recovered From Dental Calculus

**FIGURE 1:**
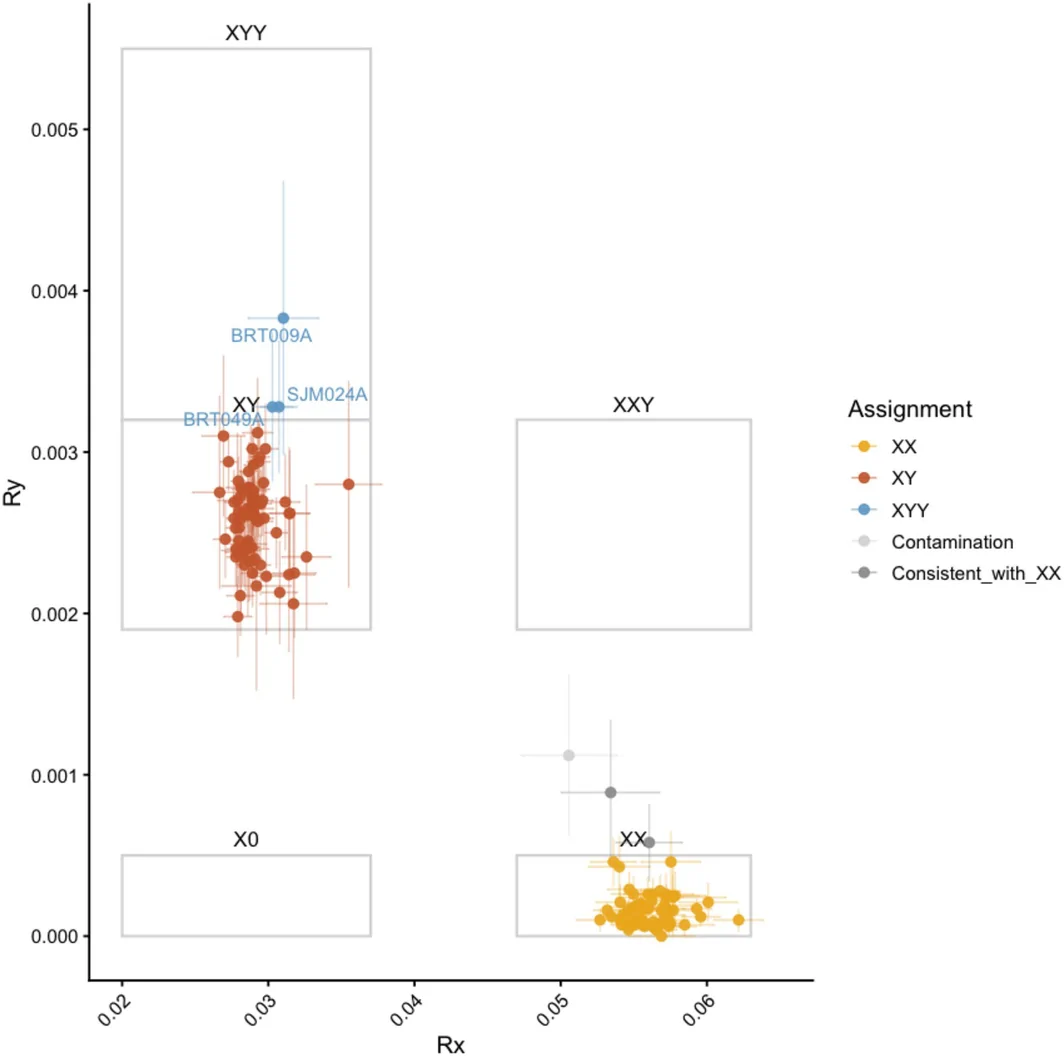
*R*_x_ and *R*_y_ estimates for the samples in the study. Each point represents one individual and the lines are the SE values.

We generated whole‐genome shotgun data for 146 individuals, all of which had an osteological sex based on macroscopic observations of the os coxae and/or skull (Table 2). Immediately following library preparation, before performing sequencing, DNA yields from dental calculus as measured by fluorometry (Qubit 2.0) ranged from 4.7 to 56 ng/μL (median 36.15 ng/μL). After sequencing, the samples yielded between 15 and 35 million DNA sequences (Table S3). The proportion of human DNA in dental calculus was low, with percentages of endogenous human content varying from 0.03 to 4.5. The average coverage depth across the human genome ranged from 0.0001 to 0.025×. Three individuals had an assignment that was XYY. Two other individuals showed an assignment “consistent with XX,” which means that the pattern reflects an XX assignment but the data is too low to give a precise prediction (Skoglund et al. 2013) (Figure 1).

**TABLE 2:** Frequencies of osteological and genetic sex estimates in the sample group.

		F	?F	?	?M	M	Unobservable	Total
Overall skeleton	Original sex estimate	54	14	1	13	64	0	146
	Pooled sex estimate	68		1		77		
Pelvis only	Original sex estimate	53	11	3	12	62	5	141
	Pooled sex estimate	64		3		74		
Skull only	Original sex estimate	46	21	23	28	25	3	143
	Pooled sex estimate	67		23		53		

Genetic sex	XX	XX?	XY	XYY	Contaminated	Total
	66	2	74	3	1	146

Abbreviations: ?, intermediate; ?F, possible female, F, female; ?M, possible male; M, male, U, unobservable.

The three individuals with a genetic sex estimate of XYY, an individual with DNA contamination, and the individuals with a genetic sex “consistent with XX” were removed from the sample before tests of accuracy were applied, producing a total sample size of 140 individuals. It should be noted, however, that all three individuals with a genetic sex estimate of “XYY” demonstrated a pelvic morphology score of “male,” while results from the cranium only were mixed.

### 4.2 Intraobserver Error Testing of Osteological Analysis

An assessment of the agreement of all data points recorded in original osteological analysis and subsequent intraobserver error testing presented with a percentage accuracy of 77.5% agreement between all data points and a kappa coefficient of “Substantial” (*κ* = 0.675) (Table 3). When scores of 1 and 2 were combined into a “female” and scores 4 and 5 into a “male” group, to test the accuracy of attributing an individual/trait to the same sex category, percentage accuracy increased to 85.3%, with a kappa coefficient of 0.766.

**TABLE 3:** Results of intraobserver error testing.

		Observation 2										
		Score frequency						Osteological sex attribution frequency				
		1	2	3	4	5	Unob.		Female	Inter.	Male	Unob.
**Observation 1**	**1**	188	50	4	2	0	26	**Female**	310	25	4	35
	**2**	20	52	21	1	1	9	**Indet**.	16	29	8	4
	**3**	1	15	29	8	0	4	**Male**	4	18	66	2
	**4**	0	4	14	16	1	0	**Unob**.	16	5	6	426
	**5**	0	0	4	5	44	2					
	**Unob**.	9	7	5	5	1	426					
% Accuracy		77.5%						85.3%				
(n/N)		(755/974)						(831/974)				
Kappa value		0.675						0.766				
Standard error		0.018						0.017				
Confidence interval		Lower	0.639					0.733				
		Upper	0.710					0.799				

Abbreviations: Inter = intermediate; Unob = unobservable.

### 4.3 Overall Sex Estimates

All individuals assessed for genetic sex were given an osteological sex estimate. Sixty‐eight individuals were allocated an osteological sex estimation of probable female/possible female, 77 an osteological sex of probable male/possible male, and one individual was determined to be intermediate for osteological sex (see Table 2). It should be noted that the skeletons used here were generally exceptionally well preserved and highly complete, allowing a large number of traits to be used to establish a sex estimate, potentially lowering the number of individuals allocated to the intermediate category.

**FIGURE 2:**
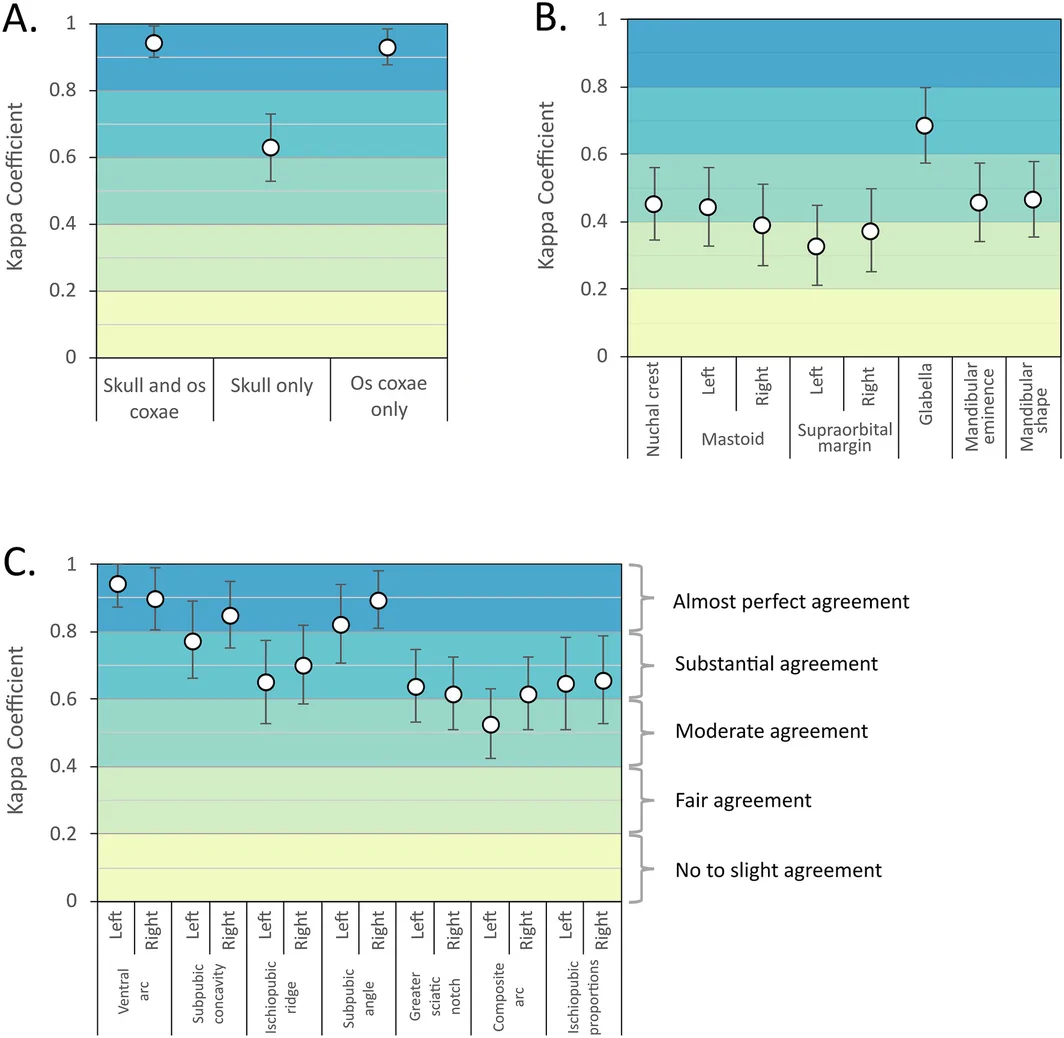
Kappa coefficient values and 95% confidence intervals in (A) different regions of the skeleton, (B) individual traits of the skull, and (C) individual traits of the os coxae.

Agreement between os coxae derived osteological sex estimates and genetic sex estimates was highly accurate at 97.8%, with a kappa coefficient of 0.942, or “near perfect” agreement (Table 4 and Figure 2A). Use of the skull only to estimate osteological sex presented with greater error, with only 79.0% agreement between osteological and genetic sex estimation, with a kappa coefficient of 0.637 (“substantial” agreement). Overall, when osteological sex was estimated using the skull and os coxa combined, allocation of osteological sex and genetic sex were in near perfect agreement, at 97.9%, with a kappa coefficient of 0.957. Osteological sex was not in agreement with genetic sex in only 3 out of the 140 individuals who had both cranial and os coxa traits observable. This is likely due to the fact that traits of the os coxae were given precedence over those of the skull when allocating overall osteological sex. It appears only the cranial observations from one individual with an intermediate pelvis allowed for additional accurate categorization of osteological sex.

**TABLE 4:** Percentage agreement between osteological sex estimation, using traits of the os coxa, the skull, and the os coxa and skull combined, and genetic sex estimation.

Skeletal region	Osteological sex estimation	Genetic sex estimation		Total correct	Total intermediate	Total incorrect	% Accuracy (n/N)^a^	Raw % accuracy (n/N)	Kappa coefficient	Standard error	95% confidence interval	
		Female	Male								Lower	Upper
Skull and os coxae	Female	65	1	137	1	2	98.7% (137/139)	97.9% (137/140)	0.957	0.024	0.910	1.004
	Male	1	72									
	Intermediate	0	1									
	Unobservable	0	0									
Skull only	Female	59	6	109	21	8	93.2% (109/117)	79.0% (109/138)	0.637	0.052	0.535	0.739
	Male	2	50									
	Intermediate	4	17									
	Unobservable	1	1									
Os coxae only	Female	62	0	131	3	1	99.2% (131/132)	97.8% (132/135)	0.942	0.028	0.887	0.997
	Male	1	69									
	Intermediate	1	2									
	Unobservable	2	3									

*Note:* Unobservable traits excluded from agreement measurements.

^a^ This accuracy is for only individuals where a sex estimate was produced. Raw accuracy is the accuracy when taking into consideration all individuals (e.g., including those that scored intermediate).

### 4.4 Individual Trait Accuracy

Percentage agreement of individual traits of the os coxae ranged from 70.6% for the left composite arch to 97.5% for the left ventral arc (Table 5). All Kappa coefficient values for traits of the os coxa fell above the 0.6 value corresponding to “substantial agreement”, except the left composite arc, which had a kappa coefficient value of 0.529 (Figure 2C). The three traits demonstrating the greatest level of agreement with genetic sex estimation were the ventral arc, subpubic concavity, and subpubic angle.

**TABLE 5:** Percentage agreement between individual osteological sex estimation traits and genetic sex estimation.

Skeletal region	Skeletal trait	Side	Osteological sex estimation	Genetic sex estimation		Total correct	Total intermediate	Total incorrect	% Accuracy (n/N)^a^	Raw % Accuracy (n/N)	Kappa coefficient	Standard error	95% confidence interval	
				Female	Male								Lower	Upper
Skull	Nuchal crest	—	Female	51	19	84	22	19	81.6% (84/103)	67.2% (84/125)	0.452	0.056	0.342	0.562
			Male	0	33									
			Intermediate	7	15									
			Unobservable	8	7									
	Mastoid	Left	Female	45	14	85	23	19	81.7% (85/104)	66.9% (85/127)	0.443	0.060	0.325	0.561
			Male	5	40									
			Intermediate	10	13									
			Unobservable	6	7									
		Right	Female	38	11	76	26	19	80.0% (76/95)	62.8% (76/121)	0.389	0.061	0.269	0.509
			Male	8	38									
			Intermediate	10	16									
			Unobservable	10	9									
	Supraorbital margin	Left	Female	38	16	73	27	24	75.3% (73/97)	58.9% (73/124)	0.328	0.061	0.208	0.448
			Male	8	35									
			Intermediate	11	16									
			Unobservable	9	7									
		Right	Female	38	14	75	23	22	77.3% (75/97)	62.5% (75/120)	0.373	0.063	0.250	0.496
			Male	8	37									
			Intermediate	9	14									
			Unobservable	11	9									
	Glabella	—	Female	50	7	95	12	8	92.2% (95/103)	82.6% (95/115)	0.687	0.057	0.575	0.799
			Male	1	45									
			Intermediate	3	9									
			Unobservable	12	13									
	Mandibular eminence	—	Female	56	19	95	19	24	79.8% (95/119)	68.8% (95/138)	0.457	0.059	0.341	0.573
			Male	5	39									
			Intermediate	5	14									
			Unobservable	0	2									
	Mandibular shape	—	Female	38	6	85	28	13	86.7% (85/98)	67.5% (85/126)	0.465	0.057	0.353	0.577
			Male	7	47									
			Intermediate	13	15									
			Unobservable	8	6									
Pelvis	Ventral arc	Left	Female	29	0	78	1	1	98.7% (78/79)	97.5% (78/80)	0.947	0.037	0.874	1.02
			Male	1	49									
			Intermediate	0	1									
			Unobservable	36	24									
		Right	Female	33	0	77	2	2	97.5% (77/79)	95.1% (77/81)	0.902	0.047	0.810	0.994
			Male	2	44									
			Intermediate	0	2									
			Unobservable	31	28									
	Subpubic concavity	Left	Female	26	0	78	8	2	97.5% (78/80)	88.6% (78/88)	0.778	0.058	0.664	0.892
			Male	2	52									
			Intermediate	7	1									
			Unobservable	31	21									
		Right	Female	35	2	87	4	1	98.9% (87/88)	92.6% (87/94)	0.854	0.051	0.754	0.954
			Male	1	52									
			Intermediate	4	0									
			Unobservable	26	20									
	Ischiopubic ridge	Left	Female	31	3	73	15	3	96.1% (73/76)	80.2% (73/91)	0.654	0.063	0.531	0.777
			Male	0	42									
			Intermediate	4	11									
			Unobservable	31	18									
		Right	Female	39	4	80	12	4	95.2% (80/84)	83.3% (80/96)	0.704	0.060	0.586	0.822
			Male	0	41									
			Intermediate	3	9									
			Unobservable	24	20									
	Subpubic angle	Left	Female	24	0	82	3	4	95.3% (82/86)	92.1% (82/89)	0.826	0.059	0.710	0.942
			Male	4	58									
			Intermediate	3	0									
			Unobservable	35	16									
		Right	Female	37	0	91	3	2	97.8% (91/93)	94.8% (91/96)	0.897	0.043	0.813	0.981
			Male	2	54									
			Intermediate	3	0									
			Unobservable	24	20									
	Greater sciatic notch	Left	Female	52	6	101	18	8	92.7% (101/109)	79.5% (101/127)	0.642	0.055	0.534	0.750
			Male	2	49									
			Intermediate	6	12									
			Unobservable	6	7									
		Right	Female	53	5	98	20	8	92.5% (98/106)	77.8% (98/126)	0.618	0.055	0.510	0.726
			Male	3	45									
			Intermediate	4	16									
			Unobservable	6	8									
	Composite arch	Left	Female	51	4	89	31	6	93.7% (89/95)	70.6% (89/126)	0.529	0.052	0.427	0.631
			Male	2	38									
			Intermediate	9	22									
			Unobservable	4	10									
		Right	Female	48	3	100	20	8	92.6% (100/108)	78.1% (100/128)	0.621	0.055	0.513	0.729
			Male	5	52									
			Intermediate	9	11									
			Unobservable	4	8									
	Ischiopubic proportions	Left	Female	17	0	53	12	1	98.1% (53/54)	80.3% (53/66)	0.650	0.070	0.513	0.787
			Male	1	36									
			Intermediate	9	3									
			Unobservable	39	35									
		Right	Female	20	0	59	13	1	98.3% (59/60)	80.8% (59/73)	0.660	0.067	0.529	0.791
			Male	1	39									
			Intermediate	9	4									
			Unobservable	36	31									

*Note:* Unobservable traits excluded from agreement measurements.

^a^ This accuracy is for only individuals where a sex estimate was produced. Raw accuracy is the accuracy when taking into consideration all individuals (e.g., including those that scored intermediate).

Traits of the skull demonstrated noticeably lower agreement values than those of the os coxa (Figure 2B). Only the glabella (agreement: 82.6%; kappa coefficient: 0.687) had comparable results to pelvic traits. Percentage agreement of the other skull traits ranged from 58.9% (left supraorbital margin) to 68.8% (mandibular eminence). Kappa coefficient values for these traits fell within the “fair” to “moderate” agreement categories.

#### 4.4.1 Side

As shown in Table 4 and Figure 2C, there are some differences in accuracy rates between the left and right side for some individual traits, but none reached statistical significance (see Table S5), with only the greater sciatic notch trending towards statistical significance (*χ* ^2^(1) = 3.085, *p* = 0.079, *N* = 271). However, in four of the five pelvic traits with notable differences in accuracies between the left and right side, the left showed lower accuracy.

#### 4.4.2 Sex Biases in Score Distributions

**FIGURE 3:**
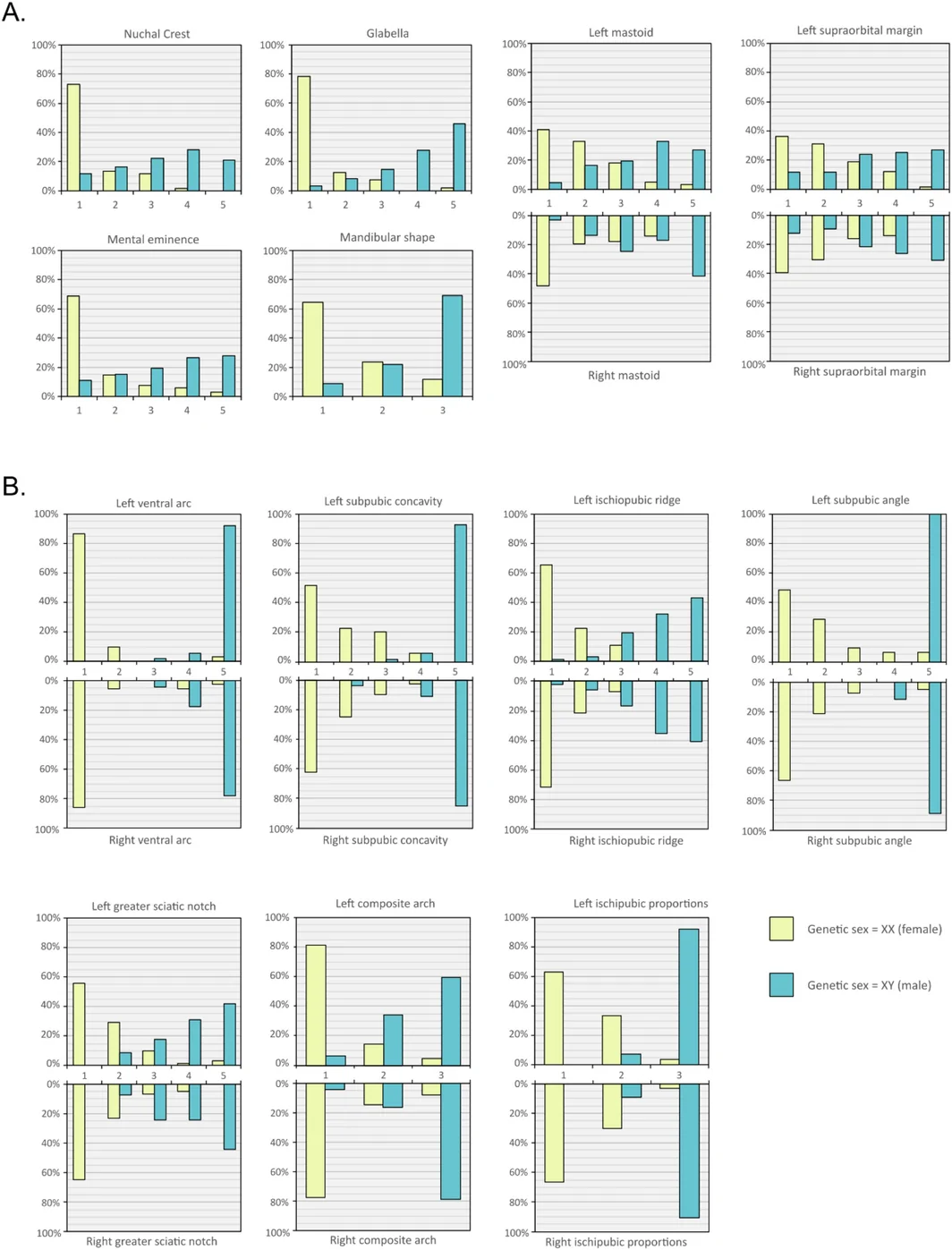
The percentage of each genetic sex category allocated to each skeletal trait grade in (A) the region of the skull, and (B) the region of the os coxa.

Table 6 outlines the individual trait accuracies for genetically estimated males and females when score “3” is taken as an inaccurate estimate. From Table 6 and Figure 3, it is possible to see that a number of trends are evidenced: features that have skewed scores (e.g., where there is a higher proportion of scores allocated to one genetic sex category) leading to a sex bias, features with evenly distributed scores across categories, and features where the score distribution is U shaped.

**TABLE 6:** The percentage accuracy of each trait according to genetically sexed male and female groups and degree of sex bias in result accuracy.

Skeletal region	Skeletal trait	Side	Score proportions: genetically sexed female					Score proportions: genetically sexed male					% raw accuracy (n/N)			Sectioning point^a^				
			1	2	3	4	5	1	2	3	4	5	Female	Male	Bias	1	2	3	4	5
Skull	Nuchal crest	—	72.9% (43/59)	13.6% (8/59)	11.9% (7/59)	1.7% (1/59)	0% (0/59)	11.9% (8/67)	16.4% (11/67)	22.4% (15/67)	28.4% (19/67)	20.9% (14/67)	86.4% (51/59)	49.3% (33/67)	37.1	F	M			
	Mastoid	Left	41.0% (25/61)	32.8% (20/61)	18.0% (11/61)	4.9% (3/61)	3.3% (2/61)	4.5% (3/67)	16.4% (11/67)	19.4% (13/67)	32.8% (22/67)	26.9% (18/67)	73.8% (45/61)	59.7% (40/67)	14.1	F		M		
		Right	48.2% (27/56)	19.6% (11/56)	17.9% (10/56)	14.3% (8/56)	0% (0/56)	3.1% (2/65)	13.8% (9/65)	24.6% (16/65)	16.9% (11/65)	41.5% (27/65)	67.9% (38/56)	58.5% (38/65)	9.4	F		M		
	Supraorbital margin	Left	36.2% (21/58)	31.0% (18/58)	19.0% (11/58)	12.1% (7/58)	1.7% (1/58)	11.9% (8/67)	11.9% (8/67)	23.9% (16/67)	25.4% (17/67)	26.9% (18/67)	67.2% (39/58)	52.2% (35/67)	15	F		M		
		Right	39.3% (22/56)	30.4% (17/56)	16.1% (9/56)	14.3% (8/56)	0% (0/56)	12.3% (8/65)	9.2% (6/65)	21.5% (14/65)	26.2% (17/65)	30.8% (20/65)	69.6% (39/56)	56.9% (37/65)	17.4	F		M		
	Glabella	—	78.2% (43/55)	12.7% (7/55)	7.3% (4/55)	0% (0/55)	1.8% (1/55)	3.3% (2/61)	8.2% (5/61)	14.8% (9/61)	27.9% (17/61)	45.9% (28/61)	90.9% (50/55)	73.8% (45/61)	17.1	F		M		
	Mental eminence	—	68.7% (46/67)	14.9% (10/67)	7.5% (5/67)	6.0% (4/67)	3.0% (2/67)	11.1% (8/72)	15.3% (11/72)	19.4% (14/72)	26.4% (19/72)	27.8% (20/72)	83.6% (56/67)	54.2% (39/72)	29.4	F	M			
	Mandibular shape	—	64.4% (38/59)	—	23.7% (14/59)	—	11.9% (7/59)	8.8% (6/68)	—	22.1% (15/68)	—	69.1% (47/68)	64.4% (38/59)	69.1% (47/68)	−4.7	F			M	
Os coxae	Ventral arc	Left	86.7% (26/30)	10.0% (3/30)	0% (0/30)	0% (0/30)	3.3% (1/30)	0% (0/50)	0% (0/50)	2.0% (1/50)	6.0% (3/50)	92.0% (46/50)	96.7% (29/30)	98.0% (49/50)	−1.3	F		M		
		Right	86.1% (31/36)	5.6% (2/36)	0% (0/36)	5.6% (2/36)	2.8% (1/36)	0% (0/46)	0% (0/46)	4.3% (2/46)	17.4% (8/46)	78.3% (36/46)	91.7% (33/36)	95.7% (44/46)	−4	F		M		
	Subpubic concavity	Left	51.4% (18/35)	22.9% (8/35)	20.0% (7/35)	5.7% (2/35)	0% (0/35)	0% (0/53)	0% (0/53)	1.9% (1/53)	5.7% (3/53)	92.5% (49/53)	74.3% (26/35)	98.1% (52/53)	−23.8	F			M	
		Right	62.5% (25/40)	25.0% (10/40)	10.0% (4/40)	2.5% (1/40)	0% (0/40)	0% (0/54)	3.7% (2/54)	0% (0/54)	11.1% (6/54)	85.2% (46/54)	87.5% (35/40)	96.3% (52/54)	−8.8	F			M	
	Ischiopubic ridge	Left	65.7% (23/35)	22.9% (8/35)	11.4% (4/35)	0% (0/35)	0% (0/35)	1.8% (1/56)	3.6% (2/56)	19.6% (11/56)	32.1% (18/56)	42.9% (24/56)	88.6% (31/35)	75.0% (42/56)	13.6	F		M		
		Right	71.4% (30/42)	21.4% (9/42)	7.1% (3/42)	0% (0/42)	0% (0/42)	1.9% (1/54)	5.6% (3/54)	16.7% (9/54)	35.2% (19/54)	40.7% (22/54)	92.9% (39/42)	75.9% (41/54)	17	F		M		
	Subpubic angle	Left	48.4% (15/31)	29.0% (9/31)	9.7% (3/31)	6.5% (2/31)	6.5% (2/31)	0% (0/58)	0% (0/58)	0% (0/58)	0% (0/58)	100% (58/58)	77.4% (24/31)	100% (58/58)	−22.6	F			M	
		Right	66.7% (28/42)	21.7% (9/42)	7.1% (3/42)	0% (0/42)	4.8% (2/42)	0% (0/53)	0% (0/53)	0% (0/53)	11.3% (6/53)	88.7% (47/53)	88.1% (37/42)	100% (53/53)	−11.9	F			M	
	Greater sciatic notch	Left	55.7% (34/61)	29.5% (18/61)	9.8% (6/61)	1.6% (1/61)	3.3% (2/61)	0% (0/67)	9.0% (6/67)	17.9% (12/67)	31.3% (22/67)	41.8% (28/67)	85.2% (52/61)	73.1% (50/67)	12.1	F		M		
		Right	65.0% (39/60)	23.3% (14/60)	6.7% (4/60)	5.0% (3/60)	0% (0/60)	0% (0/66)	7.6% (5/66)	24.2% (16/66)	24.2% (16/66)	43.9% (29/66)	88.3% (53/60)	68.2% (45/66)	20.1	F		M		
	Composite arch	Left	81.0% (51/63)	—	14.3% (9/63)	—	4.8% (3/63)	6.3% (4/64)	—	34.4% (22/64)	—	59.4% (38/64)	81.0% (51/63)	59.4% (38/64)	21.6	F		M		
		Right	77.4% (48/62)	—	14.5% (9/62)	—	8.1% (5/62)	4.5% (3/66)	—	16.7% (11/66)	—	78.8% (52/66)	77.4% (48/62)	78.8% (52/66)	−1.4	F		M		
	Ischiopubic proportions	Left	63.0% (17/27)	—	33.3% (9/27)	—	3.7% (1/27)	0% (0/39)	—	7.7% (3/39)	—	92.3% (36/39)	63.0% (17/27)	92.3% (36/39)	−29	F			M	
		Right	66.7% (20/30)	—	30.0% (9/30)	—	3.3% (1/30)	0% (0/43)	—	9.3% (4/43)	—	90.7% (40/43)	66.7% (20/30)	90.7% (40/43)	−24	F			M	

*Note:* In the calculation of percentage raw accuracy, score 3 (intermediate) here is taken as incorrect.

^a^ The sectioning point is calculated according to the grade point at which the majority proportion of one genetic sex group compared to the other changes (see Walker 2008; Tallman 2019).

Most genetically sexed females were accurately attributed a score of 1 or 2 for the nuchal crest, with 11.9% a score of 3 (intermediate), and just one individual scoring a grade of 4 (possible male). This resulted in a high accuracy in the osteological sex estimation of genetically sexed female individuals using this trait (86.4%) (see Table 6). Conversely, just over a quarter of genetically sexed males were attributed a score of 1 or 2, and a further 22.4% a score of 3. Only half of genetically sexed males (49.3%) scored in the “male” part of the spectrum, suggesting a distinct sex bias in accuracy for this skeletal feature. The glabella and, to some degree, the mental eminence show a similar pattern, whereby there is a high accuracy of attributing a score of 1 (78.2% and 68.7%, respectively) or score 2 (12.7% and 14.9%, respectively) to genetically sexed females. Few genetically sexed females scored as intermediate, or inaccurately into the male score range for either trait (1.8% and 9%, respectively). The scores for genetically sexed males, however, were more equally spread across all categories, especially for the mental eminence, resulting in a much lower degree of accuracy in men (54.2%).

Similar trends were also observed in the traits of the os coxae, although these tended to have higher accuracies for genetically sexed males than females. When accuracy was assessed in genetically sexed males according to side, 90% of observations for the left subpubic concavity, the left subpubic angle, the left composite arch, and both left and right ischiopubic proportions were attributed to the highest (i.e., “most male”) category. In genetically sexed males, the right composite arch presented with a notably higher percentage accuracy (78.8%) when compared to the left side (59.4%). The scores for genetically sexed females for the subpubic concavity and subpubic angle demonstrated a more even distribution across the score categories. The left composite arch was more accurate for genetically sexed females (81.0%) compared to males (59.4%), with a higher proportion of genetically sexed males scoring a grade 3 (34.4% in males; 14.3% in females). However, this trend was not present in the right composite arch.

Multiple traits had similar accuracies in genetically sexed males and females, but showed a higher degree of crossover between male and female categories and high counts for intermediate traits (score 3). This included the mastoids, the supraorbital margins, the left sciatic notch and to some degree the mandibular shape (see Table 6). This suggests lower dimorphism in these traits in comparison to the originating sample. A few traits showed a high degree of dimorphism between genetically sexed males and females, with little crossover and low counts of intermediate traits. These included the ventral arcs, the right subpubic concavity, right subpubic angle, and right composite arch.

### 4.5 Regression Modeling

Binary logistic regression using a backwards likelihood ratio step‐wise elimination was used to determine the combination of traits with the greatest classification accuracies with a view to improving future sex estimations. The combination of skeletal traits, as well as the combination of pelvic traits, led to a perfect fit model. Table 7 presents the most accurate combinations of cranial traits and pelvic traits separately, and includes regression equations for weighting the accuracy of different traits in combination. All cranial traits demonstrated the same or very similar level of classification accuracy using LOOCV. The glabella demonstrated the greatest classification accuracy at 92.6%. In combination, the mastoid and glabella demonstrated a high level of classification accuracy (90.6%). Inclusion of other traits did nothing to improve this classification accuracy.

**TABLE 7:** Outcomes of binary logistic regression with backward step‐wise elimination using likelihood ratio.

Skeletal region	Skeletal trait	Binary logistic regression										LOOCV classification accuracies (%)		
		*N* (female)	*N* (male)	*χ* ^2^	df	*P*	Nagelkerke *R* ^2^	original classification accuracies (%)			Regression equation (*y*)^a^			
								Female	Male	Combined		Female	Male	Combined
Skull	NC	58	67	69.929	1	< 0.001	0.572	87.9	71.6	79.2	−3.155 + 1.526 (NC)	87.9	71.6	79.2
	MA	61	70	55.857	1	< 0.001	0.464	75.4	80.0	77.9	−3.108 + 1.184 (MA)	75.4	80.0	77.9
	SM	60	70	33.284	1	< 0.001	0.302	66.7	77.1	72.3	−2.189 + 0.836 (SM)	66.7	77.1	72.3
	GL	54	61	100.084	1	< 0.001	0.776	92.6	88.5	90.4	−4.545 + 1.900 (GL)	90.4	88.5	92.6
	ME	66	72	61.229	1	< 0.001	0.478	84.8	73.6	79.0	−2.579 + 1.118 (ME)	84.8	73.6	79.0
	MS	60	68	58.445	1	< 0.001	0.496	65.5	91.2	79.4	−3.672 + 1.872 (MS)	65.5	91.2	79.4
	NC, MA, SM, GL, ME, MS	43	53	89.519	6	< 0.001	0.812	93.0	88.7	90.6	−6.404 + 0.679 (NC) + 0.377 (MA) + 0.168 (SM) + 1.133 (GL) + 0.196 (ME) + 0.271 (MS)	90.7	88.6	89.5
	NC, MA, GL, ME, MS	43	53	89.348	5	< 0.001	0.811	93.0	88.7	90.6	−6.202 + 0.643 (NC) + 0.417 (MA) + 1.178 (GL) + 0.155 (ME) + 0.348 (MS)	93.0	88.7	90.6
	NC, MA, GL, MS	43	53	89.153	4	< 0.001	0.810	95.3	88.7	91.7	−6.172 + 0.629 (NC) + 0.456 (MA) + 1.217 (GL) + 0.423 (MS)	93.0	88.7	90.6
	NC, MA, GL	43	53	88.577	3	< 0.001	0.806	95.3	88.7	91.7	−5.867 + 0.711 (NC) + 0.557 (MA) + 1.278 (GL)	90.7	88.7	89.6
	NC, GL	43	53	86.177	2	< 0.001	0.793	93.0	88.7	90.6	−5.867 + 0.868 (NC) + 1.428 (GL)	93.0	88.7	90.6
Os coxae	VA	40	55	114.173	1	< 0.001	0.940	97.5	100	98.9	−7.170 + 2.412 (VA)	97.5	96.4	96.8
	SC	46	63	116.249	1	< 0.001	0.882	95.7	95.2	95.4	−7.838 + 2.349 (SC)	95.7	95.2	95.4
	RR	46	63	103.684	1	< 0.001	0.825	87.0	95.2	91.7	−6.414 + 2.477 (RR)	87.0	95.2	91.7
	SA	45	65	130.850	1	< 0.001	0.938	95.6	100	98.2	−94.615 + 19.619 (SA)	95.6	100	98.2
	SN	63	71	112.657	1	< 0.001	0.759	87.3	91.5	89.6	−5.582 + 2.082 (SN)	87.3	91.5	89.6
	CA	64	70	97.286	1	< 0.001	0.689	81.3	94.3	88.1	−5.342 + 3.004 (CA)	81.3	94.3	88.1
	IP	33	52	83.096	1	< 0.001	0.846	97.0	90.4	92.9	−10.571 + 4.830 (IP)	97.0	90.4	92.9
	VA, SC, RR, SA, SN, CA, IP	24	43	87.419	7	< 0.001	1.000	100	100	100	−106.199 + 10.925 (VA) + 4.396 (SC) + −2.676 (RR) + −2.506 (SA) + 4.492 (SN) + 15.314 (CA) + 11.424 (IP)	91.7	97.7	95.5
	VA, SC, RR, SN, CA, IP	24	43	87.419	6	< 0.001	1.000	100	100	100	−109.214 + 10.857 (VA) + 3.891 (SC) + −2.245 (RR) + 4.195 (SN) + 14.198 (CA) + 9.497 (IP)	91.7	97.7	95.5
Os coxae	VA, SC, SN, CA, IP	24	43	87.419	5	< 0.001	1.000	100	100	100	−108.067 + 10.744 (VA) + 1.949 (SC) + 6.323 (SN) + 12.534 (CA) + 7.996 (IP)	91.7	100	97.0
	VA, SN, CA, IP	24	43	87.419	4	< 0.001	1.000	100	100	100	−113.062 + 11.636 (VA) + 8.571 (SN) + 11.407 (CA) + 8.952 (IP)	95.8	100	98.5
	VA, SN, CA	24	43	87.419	3	< 0.001	1.000	100	100	100	−151.427 + 16.937 (VA) + 16.394 (SN) + 17.750 (CA)	91.7	97.7	95.6

*Note:* Classification accuracies according to application of the test on entire sample, and using leave‐one‐out‐cross‐validation (LOOCV). Regression equation: $-5.867+0.868\left(3\right)+1.428\left(5\right)=3.862$. Probability female (*pf*): $\mathrm{pf}=\frac{1}{1+3.862^{-2.71828}}=0.0248$. Probability male (*pm*): pm=1−0.0248=0.975.

^a^ Example of the application of regression and probability equations with a nuchal crest score of 3 and a glabella score of 4.

For pelvic traits, all models presented with a high level of LOOCV classification accuracy, ranging from 88.1% (composite arch) to 98.2% (subpubic angle). In combination, traits demonstrated a 100% accuracy in the original models, with slight reduction (up to 4.5%) in the LOOCV classification accuracy, mostly affecting female classification. The combination of traits with the greatest classification accuracy was the ventral arc, greater sciatic notch, composite arch, and ischiopubic proportions at 98.5%.

### 4.6 Misclassifications

There were only three individuals that had misclassifications of sex overall using a simple scoring method, likely due to the high levels of completeness and extremely high accuracy of the os coxae. One was a female classified as a male, one was a male classified as a female, and one male classified as intermediate. When assessing the os coxae estimates, only four individuals were misclassified including a female with a male estimate, and a female with an intermediate estimate and two males with intermediate estimates. However, the vast majority of mismatches were from the skull only estimates, where 25 individuals had a mismatch with the genetic sex estimate (Table 8). Two genetically assessed females had male skull estimates, whereas 6 males had female estimates. Most significantly, 17 males had intermediate scores, showing the impact of population‐specific lower male robusticity highlighted by the distribution of scores and the sectioning point demarcations in Table 6.

**TABLE 8:** All individuals where either overall skull pelvic, or whole skeleton osteological sex estimation did not match the genetic sex estimate.

Sk. no.	Osteological sex	Genetic sex	Age	NC	MS L	MS R	SM L	SM R	GL	ME	MS	Skull	VA L	VA R	SC L	SC R	RR L	RR R	SA L	SA R	SN L	SN R	CA L	CA R	IP L	IP R	Pelvis
BH 2701	Female?	Female	YA	1	3	3	3	—	1	1	1	**1**	2	2	4	3	2	2	5	5	4	4	1	1	3	3	**3**
BH 150	Female	Female	YA	—	2	4	5	4	—	2	1	**3**	—	—	—	—	—	—	—	—	2	1	1	1	—	—	**1**
BH 2163	Female?	Female	YA	3	—	1	2	2	3	2	3	**3**	—	—	—	—	—	—	—	—	1	—	1	1	—	—	**1**
SJ 128466	Female?	Female	YA	2	5	4	2	2	1	5	3	**4**	1	1	1	1	1	1	2	2	1	1	3	3	2	2	**1**
BH 539	Female?	Female	MA	3	4	3	2	—	1	2	—	**3**	—	—	—	—	—	—	—	—	—	2	2	1	—	—	**2**
BH 771	Male?	Female	MA	3	5	—	4	4	5	2	3	**4**	2	4	4	4	3	3	5	5	3	3	2	2	—	—	**4**
SJ 115409	Female?	Male	Unk.	3	2	2	2	2	1	1	2	**2**	—	—	—	—	—	—	—	—	—	3	—	—	—	—	—
BH 1448	Male?	Male	YA	1	2	1	1	1	1	3	1	**1**	—	—	—	5	—	3	—	5	4	4	3	3	—	3	**4**
SJ 170106	Male?	Male	YA	2	3	2	1	1	2	3	2	**2**	—	—	5	5	1	1	5	5	3	3	2	1	3	3	**4**
BH 1943	Male	Male	YA	1	2	1	3	2	5	3	1	**2**	5	—	5	—	5	—	5	—	4	3	3	3	2	—	**5**
BH 407	Male	Male	YA	1	—	—	3	3	5	1	2	**3**	4	—	5	—	4	—	5	—	4	—	3	—	3	—	**5**
SJ 112288	Male?	Male	YA	2	—	3	3	3	5	—	—	**3**	5	5	—	2	2	2	5	4	3	3	2	3	—	3	**4**
SJ 135247	Male?	Male	YA	2	2	3	3	3	3	2	3	**3**	—	—	5	5	3	3	5	5	2	2	2	3	—	3	**4**
SJ 11024	Male	Male	MA	2	2	3	2	2	2	1	2	**2**	5	5	5	4	5	5	5	4	4	3	3	3	3	3	**5**
SJ 134219	Male	Male	MA	3	3	3	1	1	—	3	1	**3**	3	4	5	5	4	4	5	5	5	5	3	3	3	3	**5**
SJ 152179	Male?	Male	MA	3	2	2	2	—	—	1	—	**2**	5	5	4	4	3	3	5	5	3	3	2	2	3	3	**4**
SJ 104287	Male	Male	MA	—	1	3	2	3	4	2	2	**3**	5	5	5	5	4	4	5	5	4	4	3	3	3	3	**5**
SJ 165175	Male	Male	MA	2	3	3	5	5	2	1	2	**3**	5	5	5	5	5	5	5	5	3	3	3	3	3	3	**5**
BH 2499	Male	Male	Ma	3	3	3	5	—	—	3	3	**3**	5	—	3	—	5	—	5	—	5	5	2	2	3	—	**5**
BH 2060	Male?	Male	MA	5	1	2	4	4	5	2	1	**3**	5	—	5	—	5	—	5	—	4	4	3	3	—	—	**4**
SJ 127315	Male	Male	MA	1	3	3	4	5	2	2	3	**3**	5	5	5	4	4	4	5	4	5	5	3	3	3	3	**5**
SJ 137039	Male	Male	MA	2	3	3	4	4	5	1	3	**3**	5	5	—	2	—	3	5	4	4	4	2	3	3	3	**5**
SJ 138181	Male	Male	MA	2	2	3	3	2	4	4	3	**3**	5	5	5	5	3	4	5	5	3	3	3	3	3	3	**5**
SJ 127385	Male	Male	MA	2	4	5	1	1	3	1	3	**3**	5	5	5	5	4	3	5	5	3	3	2	3	3	3	**5**
BH 2166	Male	Male	MA	—	2	—	—	—	—	3	3	**3**	5	5	5	5	3	3	5	5	5	5	3	3	—	3	**5**
BH 800	Male	Male	MA	4	5	5	5	—	5	4	3	**5**	—	—	—	—	—	—	—	—	4	—	2	—	—	—	**3**
SJ 116031	Interm.	Male	MA	5	4	4	2	2	3	2	1	**3**	5	—	—	—	3	—	5	—	3	3	2	2	—	—	**3**
BH 2026	Male	Male	OA	5	2	3	3	3	3	2	—	**3**	4	4	5	5	3	3	5	5	5	5	3	3	—	3	**5**
BH 179	Male	Male	OA	4	3	3	2	4	4	3	2	**3**	5	5	5	5	—	5	5	5	4	4	3	3	3	—	**5**
BH 1945	Male	Male	OA	1	5	5	4	5	—	2	2	**3**	5	5	4	4	4	4	5	5	2	4	3	3	3	3	**5**

*Note:* Mismatches highlighted in red.

The effect of age on misclassification was tested using binary logistic regression and was found to have no impact in determining the accuracy of cranial osteological sex estimation (*χ* ^2^(2) = 0.405, *p* = 0.817, Nagelkerke *R* ^2^ = 0.005). Bolding highlights the overall regional scores (skull or pelvis).

## 5 Discussion

### 5.1 Accuracy of Osteological Sex Estimation in British Post‐Medieval Individuals

This research aimed to assess the accuracy of skeletal morphological features commonly scored to estimate biological sex in human skeletal remains in a majority post‐medieval sample from England. Overall, the results show that when scoring a combination of traits and prioritizing results from the os coxae, the method is highly accurate; we found a 97.9% agreement between genetic sex and osteological sex estimates when using all information from the os coxae and skull. Osteological sex estimates, using combined traits from the os coxae only, presented with a 97.8% accuracy rate and, from combined traits of the skull only, an accuracy of 79.0%. These results are similar to Inskip et al. (2019), despite the fact that different observers recorded osteological sex estimation data in each study. Inskip et al. (2019) identified that overall osteological sex estimation had a 95.6% agreement with genetic sex estimation, while os coxae traits had an agreement of 91.8%, and skull traits only had an agreement of 76.7%.

The slightly lower rates in Inskip et al. (2019) might result from the difference in completeness between the two samples. The material used in this study derived from very large collections allowing us to pick highly complete individuals, whereas in Inskip et al. (2019), all available suitable individuals were used, including some more incomplete individuals. Regardless, both studies demonstrate that, from 1150 ce through to the modern period in England, there is a clear relationship between the development of certain skeletal traits and genetic sex. As such, we can be confident that scoring multiple skeletal traits, when applied to medieval and post‐medieval remains in England, can be used to accurately infer the biological sex of a skeletonised individual. However, the os coxae is clearly preferential in the allocation of an osteological sex estimate and caution should be taken if only a skull is present; in the current study, there was an approximately one in five chance of a skull being allocated to the wrong osteological sex category. Further, excluding the glabella, individual trait accuracy for the skull ranged between 59% and 69%. In particular, this research further strengthens the point made by Inskip et al. (2019) and Kjellström (2004) that we should not be estimating sex for incomplete skulls where few traits are observable using the current published sectioning points. This point is particularly pertinent to cremated remains which often rely on the observation of heat deformed and incomplete skulls (see Gonçalves et al. 2013). However, our new sectioning points in Table 6 might prove useful when only skulls are observable for inhumated English late medieval and post‐medieval remains.

Overwhelmingly, misclassifications occurred in sex estimation of the skull in males (Table 8). The younger adult males with misclassified skulls presented with none to only one male trait. This could partially be reflective of the propensity for males to develop male skull traits in later adolescence to early adulthood (Albert et al. 2007; Houston et al. 2025). It should be noted, however, that three of these six individuals presented with a score of 5 for the glabella, indicating that this trait is still reliable in younger adults.

In middle adults, a high number of male skulls were classified as intermediate due to the presentation of majority female or ambiguous traits. This likely reflects differences in male robusticity of the cranium in the sample population compared to those used for development of the original method. Binary logistic regression to assess the impact of age indicated no relationship between age and correct sex classification, although this may be due to the small sample size of incorrectly classified individuals. Other instances of misclassification could indicate human error or the presence of too few observable traits. For example, PSN120 presented with multiple male traits in the skull, but was given an overall skull estimate of intermediate, likely as a result of human error. Whereas, PSN145 and PSN596 presented with very few observable traits of an ambiguous nature in the cranium and skull, respectively, resulting in an assignation of intermediate. In the two individuals whose overall osteological sex did not match the assigned genetic sex (PSN552 and PSN623), the quality of the human DNA recovered was not notably different from the other samples assessed, and therefore likely did not play a role in this mismatch.

### 5.2 Assessing Accuracy of Individual Traits

When observing individual trait distribution scores and accuracy rates, differences in the results between the current study and those from Inskip et al. (2019) and the few other studies that detail individual trait scores (Đurić et al. 2005; Tallman 2019; Williams and Rogers 2006) emerge. Most individuals in the current study date between 1500 and 1855 CE (with only 21 individuals dating to between 1150 and 1500 CE), while the individuals assessed in Inskip et al. (2019) date between 1200 and 1600 CE. As such, slight differences in the morphological development of the skull and pelvis over time may have impacted agreement rates among different traits in each study. Further, observer biases between the different observers performing each study may have played a role.

Skeletal morphology is the summation of a multitude of factors influencing genetics over a long time‐frame and those that act at an individual level during growth and development (Ubelaker and DeGaglia 2017). Key factors include diet, environment (ibid), disease presence (Loth and Henneberg 1996), and biomechanical loading/lifestyle (Cox 2021). Furthermore, these factors likely intertwine, resulting in a great diversity in trait expression. In reference to archaeological populations, it is also possible that there might be ramifications during significant shifts in the population genetics of a group, for example, during diaspora events. As such, the degree of sexual dimorphism expressed in a population is highly diverse and community specific. As identified in our results, we found that some of the skeletal traits may be more useful for sex estimation, while others showed low accuracies or significant sex biases, indicating important differences in either the degree of sexual dimorphism or the grades across which the dimorphism is expressed compared to the populations that were used to originate the methods.

In general, the traits of the skull had the lowest accuracy, which is consistent with other similar studies (see Table S1). This is unsurprising since there is not a strong functional selection relating to biological sex acting on these traits, unlike those of the pelvis (i.e., childbirth). Skull trait development is, therefore, more likely to be influenced by environmental and social factors than the pelvic traits. It was also the skull traits that generally demonstrated the greatest degree of sex bias in the current study. Sex biases occur when a trait more accurately identifies sex for one sex category than in the other and is commonly seen in studies on sex estimation accuracy (e.g., Gupta et al. 2022; Inskip et al. 2019; Krüger et al. 2015; Patriquin et al. 2003; Tallman 2019). This can produce overall accuracy rates that are misleading. This usually results when the sectioning point between males and females (the point between two scores at which the majority allocation to one sex category shifts) differs to that of the original method. For example, in groups with generally lower robusticity, females are likely, by default, to be scored correctly, whereas males are more likely to be incorrectly scored into female categories. In our sample, this appeared to be the case for the nuchal crest, the glabella, and the mental eminence, which had greater accuracy in females. It suggests the population we examined had a lower robusticity than the original sample upon which the methods were derived.

Interestingly, this contrasts with what was identified for the Medieval Cambridge sample, where the above traits, with the addition of the mastoids and supraorbital margins, had a sex bias towards males, suggesting that females on average tended to have more robust features. This was attributed to the possibility that medieval Cambridge women, who were likely of lower status and engaged more in manual work, would have been in more physically demanding roles that increased the likelihood of developing greater robusticity in skull traits (Inskip et al. 2019). Our findings of lower skull robusticity in post‐medieval women could also reflect the range of different activity levels represented in our sample, with some being from the middling‐to‐higher classes, who would have been less likely to be engaged in physical activity. Furthermore, in the post‐medieval period, women were more likely to be employed in manufacturing or highly focused repetitive activities which may not have had a significant weight‐bearing element, as would be more likely in agricultural‐type labor.

While all traits from the os coxa were more accurate than those of the skull, we observed that the subpubic angle and ischiopubic proportions were biased towards accurately identifying males, whereas the greater sciatic notch was biased towards identifying females. It was also observed that there was a reduced accuracy for the greater sciatic notch. Unlike the skull, where there is a deep history in studying metric and non‐metric differences for the purpose of distinguishing between groups of varying biological ancestry (see Larsen 2018), there has been far less work on understanding the variable expression of pelvic morphology, even though considerable differences in the range of variability have been found between groups. Patriquin et al. (2003) attribute highly different patterns in dimorphism for the sciatic notch between South Africans of African or European heritage to both biological ancestry and also potentially to differences in body size between the groups. Recent work by Cox (2021) explored differences in the pelvic dimensions between medieval Danish collections and found significant variation in the greater sciatic notch and subpubic angle that were not wholly attributable to biological sex. Indeed, they found that body mass, to some degree, was driving pelvic shape. More recently, Wall‐Scheffler and Kurki (2023) have proposed that the overall shape of the pelvis is not ubiquitous across human groups, with significant differences in proportions and size. They also highlight that some factors act independently on different areas of the pelvis, resulting in significant diversity in pelvic morphology between groups. It appears that while obstetrics is important, especially for the lower canal areas, population history, environment and plasticity are also significant.

As it is unlikely that there are large genetic differences between our groups (although it is possible that the material from London is more diverse than Barton‐upon‐Humber given that London was the centre of the British Empire at that time, which connected populations from across the globe), other explanations must be sought for the differences in trait accuracies between the Medieval Cambridge material and the mostly post‐medieval collections assessed here. In general, estimated height between our medieval and post‐medieval groups from England is similar (see Table S4), although it would be useful to examine significant differences in body mass as measured through the femoral head or cross‐sectional bone geometry to reaffirm this. As Cox (2021) identified there might be a relationship between pelvic dimensions and activity, although it is hard to disentangle this effect from other variables; this is also something that might drive differences between the current sample group and Cambridge. Further work is needed on very well contextualized collections to ascertain the degree of variation due to body size, but more importantly, physical activity and plasticity.

For the first time, this study tested whether there were significant differences in trait accuracy between the left and right sides. Although no traits showed statistically significantly improved accuracy for one side, four of the pelvic traits had higher accuracies on the right side. While this might be random, it is worth considering how activity patterns may affect the expression of sex estimation traits. During the Industrial Revolution, one‐sided use of the legs in a repeated and sustained activity (e.g., through the use of mechanized machines, such as sewing machines and looms) may have impacted unilateral development of sex estimation traits. Additionally, the types of mechanized activities undertaken may have also differed based on the gender role of the individual, leading to differences in accuracies based on sex. Similar effects may impact changes to sex estimation traits and requires future testing.

Further, it would also be interesting to explore how the shape and traits of the pelvis may be impacted by environment and disease. For example, it is known that vitamin D deficiency can have significant impacts on the morphology of the pelvic girdle. Since individuals with extreme pathological changes related to vitamin D deficiency are treated as pathological cases and, therefore, might be excluded from studies of morphology, to our knowledge, no one has systematically assessed how sex estimation traits might be affected. Further, it is possible that vitamin D deficiency could result in subtle changes to pelvic morphology that remain visually undetected but have significant outcomes. In addition, there have been no studies that have compared the distribution of trait scores between highly mobile populations and more sedentary groups, such as those reported here. Future comparisons, particularly as more aDNA studies take place on older collections, might enable us to understand how extreme differences in biomechanical loading, as well as changes related to pathology, relate to pelvic traits.

### 5.3 Regression Modeling and Validation

The production of regression models allowed us to assess which traits and combinations thereof were the most accurate predictors of sex when taking into consideration the different influence that individual traits might have on the outcome (e.g., traits that produce more accurate classification accuracies have greater weighting in the overall model fit). Individual cranial traits demonstrated between 72% and 93% LOOCV classification accuracy, the glabella producing the best fit. However, models combining traits of the skull provided a slightly lower classification accuracy than when applying the glabella alone. Nonetheless, the application of skull trait models did produce increased classification accuracy compared to raw accuracies for individual traits, suggesting that the application of regression equations in determining the probability of sex from the cranium may improve sex estimation of post‐medieval English individuals. Models using individual traits of the os coxa ranged from 88% to 98% LOOCV classification accuracy, with models derived from combinations of traits ranging from 96% to 99% accuracy. This indicates the near perfect fit for application of these models to traits of the os coxa in estimation sex, although it should be noted that this is not much better than the simple scoring method.

It should also be noted that while the regression equation produced here provides aid in weighting various traits, and thus the assignation of sex, these models are based entirely on the interpretations of one observer. The model will only be accurate if traits are scored accurately along the same continuum of male to female morphology as applied by the current observer. Klales et al. (2012) noted reduced classification accuracies of sex from the pelvis using logistic regression in observers with less experience. The observer in the current study is highly experienced in producing osteological sex estimates in individuals from different global populations, and particularly in post‐medieval European groups. Observers who have less experience in the range of morphology in skeletal sex estimation traits among different population groups may produce trait scores that reduce the accuracy of the model.

### 5.4 The Potential for aDNA Sex Estimation Using Dental Calculus

Previous work has demonstrated that dental calculus contains both microbial and human DNA, though the latter is typically present at low proportions compared to skeletal elements (Warinner et al. 2014; Ozga et al. 2016). However, the use of human DNA extracted from dental calculus here confirms that sufficient host‐derived DNA can be preserved in dental calculus in archaeological contexts to determine genetic sex. The low endogenous human content observed here (0.03%–4.5%) and the extremely shallow average genomic coverage (0.0001–0.025×) are, therefore, consistent with established expectations for shotgun sequencing of calculus. However, confident sex assignment was possible for the majority of individuals. Specifically, 66 individuals were assigned as XX and 74 as XY, demonstrating that even ultra‐low coverage data and sequencing of libraries built from dental calculus can provide meaningful biological information. Two individuals produced assignments classified as “consistent with XX,” meaning that the ratio of X‐ to Y‐derived reads matched expectations for an XX karyotype but did not reach statistical thresholds for high‐confidence classification (Anastasiadou et al. 2024). The identification of three individuals exhibiting patterns suggestive of an XYY karyotype warrants cautious consideration and cannot be used for further interpretation. Given the extremely low coverage and wide confidence intervals in these samples, we also excluded these individuals from downstream analyses.

Overall, these results demonstrate both the feasibility and the constraints of recovering host genomic information from dental calculus. While this substrate offers a valuable alternative source of human aDNA, especially in contexts where destructive sampling of skeletal elements is restricted, interpretations derived from ultra‐low coverage shotgun data must remain conservative. Target enrichment of human DNA from ancient dental calculus could substantially improve the robustness of karyotypic inference from this material.

## 6 Conclusion

This research aimed to assess the accuracy of individual morphological traits, and the sex estimates derived from their combination, in post‐medieval British remains, using genetic sex estimates derived from dental calculus as a proxy for biological sex. Overall, the estimates derived from the os coxae traits, and the os coxae and skull traits combined, were highly accurate, although the high accuracy achieved in the combined estimate was due to the outstanding performance of the os coxae. Use of the skull for sex estimation was far less accurate; we identified significant sex biases for some traits and high rates of allocation to the intermediate group, which highlighted discordance in the pattern of sexual dimorphism between our British populations and those used to originate the methods. Nevertheless, we present new sectioning points that might prove useful to other researchers, in addition to regression equations that will provide probabilities of individuals being one sex or another, the latter of which are important for the skull. Overall, we show that there can be confidence in sex estimates derived from currently used morphological trait methods, but care should be taken when the os coxae is unobservable, and attention should be paid to traits that had sex biases in their accuracies. In addition, the benefits of using genetic data obtained as a by‐product of oral microbiome studies for sex estimation are demonstrated. This acts as an alternative approach to destructive sampling of the petrous pyramid or ear ossicles when only information about genetic sex is needed. Further work is required to assess the relationship between socioenvironmental factors and the development of sexual dimorphism in the pelvic structure, exploration of which has been neglected in comparison to the skull.

## Author Contributions

**Biancamaria Bonucci:** investigation, writing – original draft, methodology, writing – review and editing, formal analysis. **Sarah Alice Inskip:** conceptualization, investigation, funding acquisition, writing – original draft, methodology, writing – review and editing, project administration, resources. **Christiana Lyn Scheib:** writing – review and editing, resources, funding acquisition. **Anna Myfanwy Davies‐Barrett:** conceptualization, investigation, writing – original draft, writing – review and editing, methodology, validation, formal analysis.

## Funding

Funding for this research was awarded to S.A.I. UKRI‐FLF (AHRC) grant no MR/T022302/1 and a Phillip Leverhulme Prize from the Leverhulme Trust awarded to S.A.I.

## Ethics Statement

This research was undertaken following ethical guidance outlined by the British Association for Biological Anthropology and Osteoarchaeology. The research was assessed and reviewed by the ethics board at the University of Leicester (ref no 8448‐si159‐ss/ah:archaeology&ancienthistory).

## Conflicts of Interest

The authors declare no conflicts of interest.

## Supporting information


**Table S1:** Reported accuracy of morphological and biomolecular sex estimation methods.
**Table S2:** Sample composition from St James, Euston and Barton‐upon‐Humber collections.
**Table S3:** Results of the genetic analysis.
**Table S4:** Stature of males and females from St James, Barton upon Humber and the Hospital of St Johns, Cambridge.
**Table S5:** Results of Chi‐squared tests showing lack of significant difference in accuracy between left and right side traits.
**Table S6:** Details of the individuals used in the study, their genetic and morphological sex estimates and scores per morphological feature.
**Table S7:** Scores and results of intraobserver error tests for morphological traits.

**Data S1:** ajpa70328‐sup‐0002‐Table‐Legends.docx.

**Text S1:** aDNA methodology and pipeline.

## Data Availability

The data used in this study are contained within the article and its Supporting Information. The genetic data generated and analyzed during the current study are available in the ENA repository under the accession ID: PRJEB111943.
