# Supplementary material for: Comparing the Accuracy of Osteological Sex Estimation Against Genetic Sex Obtained From Human Dental Calculus in 12th–19th C. British Individuals
Source: Am J Biol Anthropol. 2026 Aug 7;190(4):e70328. doi: 10.1002/ajpa.70328 (PMC13449166; doi:10.1002/ajpa.70328)
Supplement: Supplementary file 1 [file AJPA-190-e70328-s002.docx]

**Supplementary text 1**

**aDNA methodology and pipeline**

**DNA extraction**

All subsequent steps were performed in the Ancient DNA Facility of the Core Facility of Genomics, Institute of Genomics, University of Tartu. The dental calculus samples were weighed and up to 20 mg of each sample was collected in a sterile tube. Next, 500 μl of EDTA was added to each sample and the tubes were left rotating for around 10 minutes at room temperature on a nutating mixer. The tubes were then centrifuged and the EDTA was carefully removed and stored for later testing. Then, 500 μl of fresh EDTA was added again to each sample and the tubes were incubated at room temperature on a nutating mixer for 72-144 h, with the duration adjusted based on the progress of demineralization of the matrix. After this time, 450 μL of each DNA extract was purified in large volume columns (High Pure Viral Nucleic Acid Large Volume Kit, Roche) using 10 x mL of PB buffer, 1 mL of PE buffer and 100 μL of EB buffer (MinElute PCR Purification Kit, QIAGEN) per sample. For the elution of DNA, the silica columns were transferred to collection tubes to dry and, afterwards, in 1.5 mL DNA lo-bind tubes (Eppendorf) to elute. The samples were incubated with 100 μL EB buffer at 37°C for 10 min and centrifuged at 17,949 x g for 2 min. After centrifugation, the silica columns were removed, and the samples were stored at −20°C.

**Library preparations and sequencing**

The non UDG-treated double-stranded DNA (dsDNA) libraries were prepared following established protocols [(Keller et al., 2022, 2023)](https://paperpile.com/c/SW0gyR/Xw0di+oOhcW). Three verification steps were implemented to make sure library preparation was successful and to measure the concentration of DNA in the libraries before sequencing: fluorometric quantitation (Qubit, Thermo Fisher Scientific), parallel capillary electrophoresis (Tape Station, Agilent Technologies) and qPCR (KAPA Library Quantification Kit for Illumina® platforms, Roche). Samples were sequenced using the Illumina NextSeq 500 platform (with the 150 bp paired-end method) and the NextSeq2000 platform (with the 100 bp single end method).

**Pre-processing of sequences and mapping**

Before mapping, the sequences of the adapters, indexes, and poly-G tails were cut from the ends of DNA sequences using cutadapt-2.1 [(Martin, 2011)](https://paperpile.com/c/SW0gyR/jldWs) and sequences shorter than 28 bp were also removed to avoid random mapping. For the paired-end sequences, the R1 and R2 reads were then merged using FLASH-1.2.11 [(Magoč & Salzberg, 2011)](https://paperpile.com/c/SW0gyR/cjwNF) and the sequences were then mapped to the human reference genome GRCh37 (hs37d5) with Burrows-Wheeler Aligner (BWA 0.7.17) [(Li & Durbin, 2009)](https://paperpile.com/c/SW0gyR/haObb) and command aln 0.04 with reseeding disabled.

The processed reads were aligned to the human reference genome GRCh37 (hs37d5) using BWA v0.7.17 (Li & Durbin, 2009) with the aln algorithm (0.04), disabling reseeding. Following alignment, SAM files were converted to BAM format and only reads mapping to the human genome were retained using samtools v1.9 (Li & Durbin, 2009). Data generated from different flow cell lanes were subsequently merged, and duplicates were removed using Picard v2.20.8 (Broad Institute). Indel realignment was performed with GATK v3.5 (McKenna et al., 2010), and reads with mapping quality scores below 10 were filtered out using samtools v1.9.

**aDNA authentication, contamination estimates and statistics for generated data**

We used mapDamage 2.0.6 [(Jónsson et al., 2013)](https://paperpile.com/c/SW0gyR/Tg7s5) to estimate the frequency of C to T transitions in the 5’ end of the DNA fragments. This measure is used to authenticate whether the DNA found in the sample is coherent with aDNA characteristics. The samples show on average 10,03% C→T substitutions at the 5′ ends, with values ranging from 3.5 to 23.5%. The mtDNA contamination and chrX contamination of male individuals could not be calculated due to the low coverage values of our samples. The number of final reads, average read length, average coverage and other statistics were calculated using Samtools 1.3 stats option and an in-house script.
