# Supplementary material for: Comparing the Accuracy of Osteological Sex Estimation Against Genetic Sex Obtained From Human Dental Calculus in 12th–19th C. British Individuals
Source: Am J Biol Anthropol. 2026 Aug 7;190(4):e70328. doi: 10.1002/ajpa.70328 (PMC13449166; doi:10.1002/ajpa.70328)
Supplement: Supplementary file 2 [file AJPA-190-e70328-s003.docx]

**Supplementary table headings**

**Table S1** Reported accuracy of morphological and biomolecular sex estimation methods.

**Table S2** Sample composition from St James, Euston and Barton-upon-Humber collections

**Table S3** Results of the genetic analysis

**Table S4** Stature of males and females from St James, Barton upon Humber and the Hospital of St Johns, Cambridge.

**Table S5** Results of Chi-squared tests showing lack of significant difference in accuracy between left and right side traits.

**Table S6** Details of the individuals used in the study, their genetic and morphological sex estimates and scores per morphological feature.

**Table S7** Scores and results of intraobserver error tests for morphological traits
